# Implicating the cholecystokinin B receptor in liver stem cell oncogenesis

**DOI:** 10.1152/ajpgi.00208.2023

**Published:** 2024-01-22

**Authors:** Martha D. Gay, Jack C. Drda, Wenqiang Chen, Yimeng Huang, Amal A. Yassin, Tetyana Duka, Hongbin Fang, Narayan Shivapurkar, Jill P. Smith

**Affiliations:** ^1^Department of Medicine, Georgetown University, Washington, District of Columbia, United States; ^2^Department of Oncology, Georgetown University, Washington, District of Columbia, United States; ^3^Department of Biostatistics, Bioinformatics and Biomathematics, Georgetown University, Washington, District of Columbia, United States

**Keywords:** cholecystokinin (CCK)-B receptor, liver cancer, proglumide, stem cells, tumorspheres

## Abstract

Hepatocellular carcinoma (HCC) is the fastest-growing cause of cancer-related deaths worldwide. Chronic inflammation and fibrosis are the greatest risk factors for the development of HCC. Although the cell of origin for HCC is uncertain, many theories believe this cancer may arise from liver progenitor cells or stem cells. Here, we describe the activation of hepatic stem cells that overexpress the cholecystokinin-B receptor (CCK-BR) after liver injury with either a DDC diet (0.1% 3, 5-diethoxy-carbonyl 1,4-dihydrocollidine) or a NASH-inducing CDE diet (choline-deficient ethionine) in murine models. Pharmacologic blockade of the CCK-BR with a receptor antagonist proglumide or knockout of the CCK-BR in genetically engineered mice during the injury diet reduces the expression of hepatic stem cells and prevents the formation of three-dimensional tumorspheres in culture. RNA sequencing of livers from DDC-fed mice treated with proglumide or DDC-fed CCK-BR knockout mice showed downregulation of differentially expressed genes involved in cell proliferation and oncogenesis and upregulation of tumor suppressor genes compared with controls. Inhibition of the CCK-BR decreases hepatic transaminases, fibrosis, cytokine expression, and alters the hepatic immune cell signature rendering the liver microenvironment less oncogenic. Furthermore, proglumide hastened recovery after liver injury by reversing fibrosis and improving markers of synthetic function. Proglumide is an older drug that is orally bioavailable and being repurposed for liver conditions. These findings support a promising therapeutic intervention applicable to patients to prevent the development of HCC and decrease hepatic fibrosis.

**NEW & NOTEWORTHY** This investigation identified a novel pathway involving the activation of hepatic stem cells and liver oncogenesis. Receptor blockade or genetic disruption of the cholecystokinin-B receptor (CCK-BR) signaling pathway decreased the activation and proliferation of hepatic stem cells after liver injury without eliminating the regenerative capacity of healthy hepatocytes.

Listen to this article’s corresponding podcast at https://ajpgi.podbean.com/e/got-guts-the-micro-version-implicating-the-cholecystokinin-b-receptor-in-liver-stem-cell-oncogenesis/.

## Graphical abstract

**Figure:**
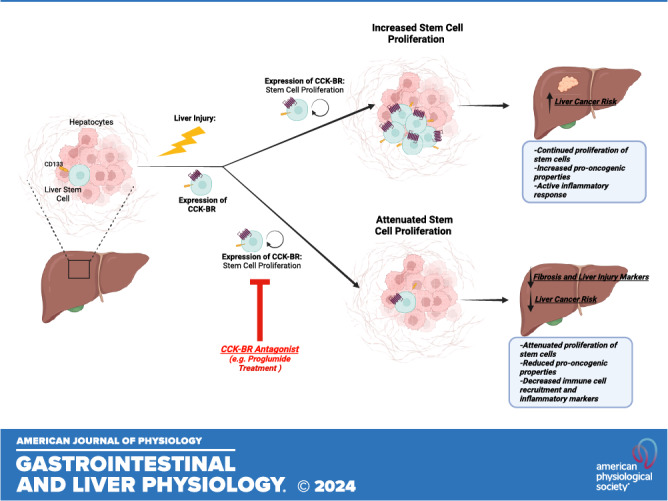

## INTRODUCTION

Hepatocellular carcinoma (HCC) is the second leading cause of cancer-related deaths worldwide (1). Although the development of HCC rarely occurs in histologically normal livers, the risk for HCC considerably increases after liver injury or chronic inflammation when normal hepatic parenchyma is replaced with fibrosis, and 90% of HCC cases arise in damaged livers with advanced fibrosis or cirrhosis (2–4). Although the cellular origin for HCC remains uncertain, it has been proposed that this cancer may arise from liver progenitor cells or stem cells (5). The liver has a remarkable ability to regenerate after injury from viral, alcoholic, nonalcoholic steatohepatitis (NASH), or resection where the hepatocytes hypertrophy and divide to restore hepatic mass and function (6). However, when the regenerative capacity of hepatocytes is severely impaired, epithelial cells with intermediate hepatocyte-cholangiocyte phenotypes emerge and expand. These cells, formerly called oval cells, are considered to be bipotential progenitors (7). It is postulated that HCC could originate from stem cells due to “maturation arrest” or “dedifferentiation” of mature cells (7); however, the mechanism that allows activated stem cells to become oncogenic is unknown.

During liver injury, reactivation of embryonic proliferative genes and receptors may occur in cells undergoing cellular reprogramming and dedifferentiation (8). One of the most frequently used cell surface markers for detecting and isolating hepatic stem cells is CD133 (CD133/prominin 1; PROM1; 9). Several studies have previously demonstrated that CD133‐positive liver stem cells have the potential for tumor initiation (10, 11). Another cell surface marker for hepatic progenitor cells is cytokeratin 19 (CK19; 12). CK19 is detected in primitive hepatic progenitor cells at 4–10 wk gestation, but it is not expressed in mature hepatocytes. However, CK19 is reexpressed after hepatic injury (12).

The cholecystokinin B receptor (CCK-BR) is not found in the normal liver but becomes expressed with liver injury (13) and it is overexpressed in HCC (14, 15); therefore, this receptor is a prime candidate implicated in liver oncogenesis. Several murine models have been developed to study liver injury including the DDC diet (0.1% 3, 5-diethoxy-carbonyl 1,4-dihydrocollidine; 16) and the choline-deficient ethionine supplemented (CDE) diet (17, 18). These dietary animal models have previously demonstrated the activation of hepatic progenitor cells and the development of HCC similar to that in human liver tissues (19). In a prior study by Tucker et al. (20), mice fed a 0.75% CDE NASH-inducing diet over 18 wk developed liver injury with fibrosis, steatosis, and inflammation, and one-third of the mice developed dysplastic nodules or HCC. However, liver injury in this study was ameliorated, and development of HCC was completely prevented in mice on this diet by concomitant administration of a CCK-BR antagonist proglumide (20). Proglumide treatment has also been reported to decrease the growth of established HCC tumors in mice (13). These data suggest that the CCK-BR is activated with liver injury and that this receptor’s expression is involved in hepatic oncogenesis.

Many types of in vitro three-dimensional (3-D) culture systems have been developed to recapitulate the in vivo growth conditions of cancer and embryonic stem cells (21) including tumorspheres (spheroids) and organoids. One feature that allows cells to form the 3-D culture systems is the “stemness” of the cells involved or the concentration of stem cells with pluripotent characteristics (21–23). These stem cells proliferate in vitro and have characteristics that resemble tumors. Injury and regeneration of the mouse adult liver result in the expression of *Lgr5+* stem cells near bile ducts (24). Hepatic stem cells express multiple *Wnt* target genes and have hallmarks of bipotential liver progenitors (24).

We hypothesized that with liver injury, there is activation of hepatic stem cells or progenitor cells that express the CCK-BR, and blockade of this receptor during hepatic injury will decrease the risk for hepatic oncogenesis. We tested the role of the CCK-BR in hepatic stem cell activation in both a pharmacologic (proglumide) model and a genetically engineered (CCK-BR-KO) murine model using both the DDC and CDE liver injury models. We found that with the liver-inducing injury diets proglumide-treated mice and transgenic CCK-BR-KO mice exhibited reduced liver inflammation and fibrosis, decreased the activation of hepatic stem cells, and the prevention of 3-D tumorsphere formation. In this work, we also show that activation of the CCK-BR signaling pathway is involved in hepatic carcinogenesis after injury by induction of a population of CD133+ stem cells that express the CCK-BR. The clinical significance of our work is that the application of CCK-BR antagonist therapy to human subjects with hepatic injury, chronic inflammation, or fibrosis with proglumide could potentially decrease the risk for the development of HCC.

## METHODS

### Animal Models

All mouse studies were performed ethically and approved by the Institutional Animal Care and Use Committee (IACUC) at Georgetown University. All mice were housed in filter top cages with five mice per cage and exposed to a 12 h light/12 h dark cycle while maintained at a constant temperature of 20°C–22°C. Mice and food were weighed weekly, and the animals received food and drink ad libitum. Four separate animal studies were performed to assess the CCK-BR’s role in liver injury. The first study was a pharmacologic study in forty male C57BL/6 mice (Charles Rivers) to examine the activation of liver stem cells and the formation of 3-D tumorspheres by the DDC diet, and whether proglumide therapy affected the oncogenic potential of the stem cells. In the second mouse study, 60 10-wk-old male C57BL/6 mice (Charles Rivers) were used to determine the duration of the DDC diet required to induce liver injury and whether proglumide could hasten recovery of the histologically damaged liver. The third experimental study utilized a transgenic mouse model selectively bred to establish a colony of mice that were CCK-BR-Knockout (CCK-BR-KO) and CCK-BR-heterozygous (25). A cryopreserved egg (Jackson Labs) that was 129-*Cckbr^tm1Kpn^*/J was inoculated into a fertile C57BL/6J female mouse to establish heterozygous pups. The pups were bred and genotyped to develop a transgenic colony. Primers for the genotyping are shown in Supplemental Table S1. In the fourth model, mice (*n* = 10) were fed a NASH-inducing CDE diet with saturated fat to determine if CCK-BR-expressing stem cells were activated in a second liver injury model.

### Treatments

The CCK receptor antagonist proglumide (Tocris) was resuspended in the animals’ drinking water (0.1 mg/mL) with an estimated amount ingested per mouse of 30 mg/kg/day, a dose we previously found effectively blocked the CCK-BR (26). Control mice were fed a standard mouse diet, Lab Diet 5001 (Purina). The DDC liver injury model group was fed a 0.1% 3,5-diethoxycarbonyl-1,4-dihydrocollidine (Sigma) supplemented diet, and the DDC “½” pellet was then manufactured by PMI Nutrition International (Lab Diet 5001 with 0.1% DDC). The choline-deficient-ethionine supplemented diet (CDE) custom high-fat diets (MP Biomedicals) contained casein as the major source of protein and lard (saturated fat) as the primary fat source. This CDE diet was modified to a 75%-CDE diet composition (18) with 25% less ethionine to avoid hemorrhagic pancreatitis associated with a 100% CDE diet.

### Measurement of Animal Weights and Food

Since these liver injury diets may result in hepatitis and weight loss, food consumption and weight were recorded in each study, and the experiment or diet was stopped if mice lost > 10% of baseline body weight.

### Biochemical Determination of Liver Injury

After each study before necropsy, blood was collected by cardiac puncture. The blood from each mouse was centrifuged, and the serum was frozen at −80°C for downstream hepatic chemistry analysis by VRL Laboratories.

### Histology Evaluation of Liver Tissues

After 4 wk on the DDC diet, mice were euthanized, and mice were necropsied, and the livers were harvested for analysis. In the reversal study mice were necropsied after 6 wk of the DDC diet followed by 4 wk of the standard chow. CDE-fed mice were necropsied, and the livers were collected for histology at *week 8*. Livers were fixed in 4% paraformaldehyde and then were embedded with paraffin. Histological tissue sections (5 µm) were reacted with Masson’s trichrome stain to analyze hepatic fibrosis for comparison with the control mice livers.

Liver sections (5 µm) from each mouse were also analyzed for the activation of hepatic stem cells with immunohistochemistry using CD133 (Prominin-1) monoclonal rat antibody (13A4), (eBioscience) at a titer of 1:50 and cytokeratin 19 (CK19) polyclonal rabbit (Novus Biologicals) at a titer of 1:200 overnight at 4°C followed by incubation with an horseradish peroxidase (HRP)-secondary antibody at room temperature. Images (*n* = 10–20) were taken of each sample from the slides using an Olympus BX61 microscope with a DP73 camera. The mean densitometry of Masson’s trichrome staining per slide was calculated using integrative density with the Image-J computer software. Images from the immunohistochemical stains were manually counted for CD133 or CK19-positive cells with the assistance of the ImageJ software cell counting tool.

### Liver Dissociation

The CD133+ stem cell isolation was adapted from Rountree et al. (27). DDC-fed mice were euthanized at 4 wk, and the abdominal cavity was wiped with 70% ethanol. The livers were excised, and the gallbladders were removed. Approximately 800 mg of the liver was sectioned and washed with DMEM supplemented with stable glutamine. The liver section was minced and then homogenized using a Miltenyi Liver dissociation kit (Miltenyi Biotec) in a gentleMACS Octo Dissociator. Liver homogenates were filtered with a MACS SmartStrainer (30 µm). The flow-through cell suspension was centrifuged at 300 *g* for 10 min. The cell pellet was resuspended in 5 mL of 1× RBC lysis buffer (ThermoFisher) and incubated for 5 min at room temperature. Lysis was halted with the addition of 20 mL 1× PBS. Cell counting and viability were assessed. If cell viability was below 90%, dead cell removal was performed with the Miltenyi dead cell removal kit. Cells were kept on ice throughout the preparation.

### CD 133+ Stem Cell Isolation and Flow Cytometry

Cell suspensions were normalized for a total of 10^7^ cells and centrifuged at 300 *g* for 10 min. The supernatant was aspirated and then resuspended in a 1:250 PE-conjugated CD133 antibody solution, 1× PBS, pH 7.2; 0.5% BSA; and 2 mM EDTA. The solution was incubated for 10 min in the dark at 4°C. The unbound primary antibody was removed by adding 1 mL of buffer and centrifuging at 300 *g* for 10 min. The cell pellet was resuspended in 80 μL buffer. Miltenyi Anti-PE MicroBeads UltraPure (20 μL) were added to the cell preparation. CD 133+ stem cells were positively selected using Miltenyi LC columns. The enriched CD133+ liver stem cell flow-through was stained with 1 μL of Alexa Fluor 488-conjugated CCK-BR antibody and 1 μL of CD45 antibody (Brilliant Violet 650). Gating was used to exclude CD45+ cells in flow analysis.

### 3D-Spheroid Formation In Vitro

Isolated liver stem cells were placed in 24-well low cell attachment plates (Nunclon Sphera 3-D culture ware, Thermo Fisher Scientific) in the presence of a ready-to-use kit including Basal Medium and Supplement Mix, 250 mL (Sigma) and 3-D Tumorsphere medium XF. Cells were seeded in appropriate suspension culture wells (a density of 10,000 cells/mL) and placed in an incubator with 5% CO_2_ and 95% humidified air at 37°C. After 5 and 10 days, photos were taken to compare the formation and growth of tumorspheres from each animal group.

### Analysis of 3-D-Tumorsphere Proliferation by MTT Assay

Tumorsphere cells from control, proglumide, DDC-fed mice, and DDC-fed with proglumide treatment were seeded at 1,000 cells/well (0.1 mL) into each well of a flat bottom low adherent 96-well plate in 3-D Tumorsphere XF phenol red-free medium. Tumorspheres were allowed to grow for 4 days in the incubator at 37°C. After the incubation period, 10 µL of MTT reagent [3-(4,5-dimethylthiazol-2-yl)-2,5-diphenyl tetrazolium bromide] 10 mg/mL was added to each well (final concentration of 0.5 mg/mL). Tumorspheres were incubated, covered in the dark for 4 h in the incubator at 37°C in 5% CO_2_ with humidified atmosphere. After 4 h, DMSO (dimethyl sulfoxide, 100 µL) was added to each well to dissolve the formazan. The plate was analyzed with the absorbance at 570 nm.

In another proliferation MTT assay, tumorspheres from DDC-fed mice were plated as above in a 96-well low adherence plate and exposed to media alone (control) or CCK (10 and 30 nM) for 4 days. MTT reagent and DMSO were then added to the wells of the plate, and percent viability was analyzed on the plate reader. In a third MTT proliferation assay, tumorspheres from the livers of control-diet-fed mice or spheroids from the liver of DDC-fed mice were plated as above in a 96-well low adherence plate and exposed to media alone or proglumide (10 nM). Effects on tumorsphere proliferation were evaluated after 4 days of treatment.

### Liver Cytokine RNA Expression

To understand differentially expressed genes regulating the hepatic inflammation in the DDC model and how the inflammation was decreased with proglumide, we conducted an array using an 84 gene RT^2^ Profiler PCR array (Qiagen; PAMM-181Z) to analyze mRNA from the DDC mouse model involved in mouse cancer inflammation and immunity cross talk. RNA was isolated from the livers of control (normal chow-fed mice); 0.1% DDC liver injury diet mice; and mice receiving the 0.1% DDC diet and proglumide treatment. Isolation was performed with the RNeasy kit (Qiagen). For the control cohort, the RNA was pooled (*n* = 4 each), and for the DDC and DDC-proglumide cohorts, two arrays were performed, each with two different RNA pools (*n* = 8 total). cDNA was synthesized using the RT2 first strand kit (Qiagen). Expression profiles of cytokines, chemokines, and other cancer inflammatory makers in the PCR array from each cohort were evaluated using an Applied Biosystems 7300 Real-Time PCR System. The results were normalized to the control cohort via the ΔΔCT method.

### RNA Sequencing

RNA from the mice’s livers was extracted and pooled as described earlier (*n* = 4). The RNA integrity and quantitation were assessed using an RNA Nano 6000 assay kit with the Bioanalyzer 2100 system (Agilent Technologies, Santa Clara, CA). Only samples with RNA integrity number (RIN) values > 8.0 were utilized for RNAseq analysis. Library preparation for transcriptome sequencing was performed by Novogene Co., Ltd. (Sacramento, CA) with 1 µg RNA per sample on a NovaSeq6000 sequencing machine. Further analysis and visualization were performed with ingenuity pathway analysis (Qiagen, Hilden, Germany) and R programming language. Differentially expressed genes were identified with a logFC between −0.5 and +0.5 with an adjusted *P* value of <0.05, correcting for false discovery via the Benjamini–Hochberg method. Canonical pathways and gene networks were identified with the ingenuity pathway knowledge base and the Database for Annotation, Visualization, and Integrated Discovery (DAVID).

### Multiplex Immunohistochemical Analysis of the Liver Microenvironment

Livers from the DDC wild-type mice treated with proglumide-supplemented drinking water, or untreated water were fixed and mounted on glass slides as described earlier. After antigen retrieval and antibody optimization, sections were stained according to the Multiplex immunohistochemistry protocol by Wang et al. (28) with the following rabbit polyclonal antibodies and titers: Ki67 (1:400; Abcam, ab15580); collagen1α1 (1:50; ThermoFisher, PA5-89281); CD68 (1:1,000, ThermoFisher, PA5-78996); and rabbit monoclonal antibodies, clones, and titers: E-cadherin (clone 24E10; 1:200, Cell Signaling, 3195); CD8 (clone D4W2Z; 1:50, Cell Signaling, 98941), and CD31 or PECAM-1 (clone, D8V9E; 1:150, Cell Signaling, 77699) for 30 min at room temperature in the Leica BOND autostainer. Sections were counterstained with an EnVision + System HRP-labeled polymer anti-rabbit (Agilent, K400311-2). Images were viewed and analyzed using PhenoChart and Inform imaging software. Nuclei were reacted with DAPI (AKOYA Biosciences, FP1490).

### Statistical Analysis

The sample size for the mouse studies was calculated based on efficacy in prior trials that showed *n* = 6–10 mice/treatment group were needed to demonstrate statistical significance between the cohorts. Data was analyzed using GraphPad Prism version 10.0 with means between treatment groups compared using Student’s *t* test with Bonferroni correction for multiple comparisons to controls or two-way ANOVA with Tukey test correction for multiple comparisons. Real-time PCR results were analyzed using a Student’s *t* test on the normalized mean ΔCT (the difference between the cycle counts of the gene of interest minus the count of an endogenous control) values for each group, with Bonferroni corrections applied to adjust for multiple comparisons. Significance was set at 95% confidence intervals and *P* value of <0.05. The RNA sequencing data were analyzed with Qiagen ingenuity pathway analysis (IPA) for bioinformatics analysis. IPA uses a network generation algorithm to segment the network map between molecules into multiple networks and assign scores for each network. The network overlap function was used to generate a network that connects pathways that had overlapping genes.

## RESULTS

### CCK-BR Blockade or Genetic Knockout Ameliorates Liver Injury

The hallmark of liver injury is the elevation of hepatic transaminases which includes the measurement of serum alanine aminotransferase (ALT) and aspartate aminotransferase (AST). Synthetic hepatic function is assessed by the measurement of serum albumin and total bilirubin.

**Figure 1.**
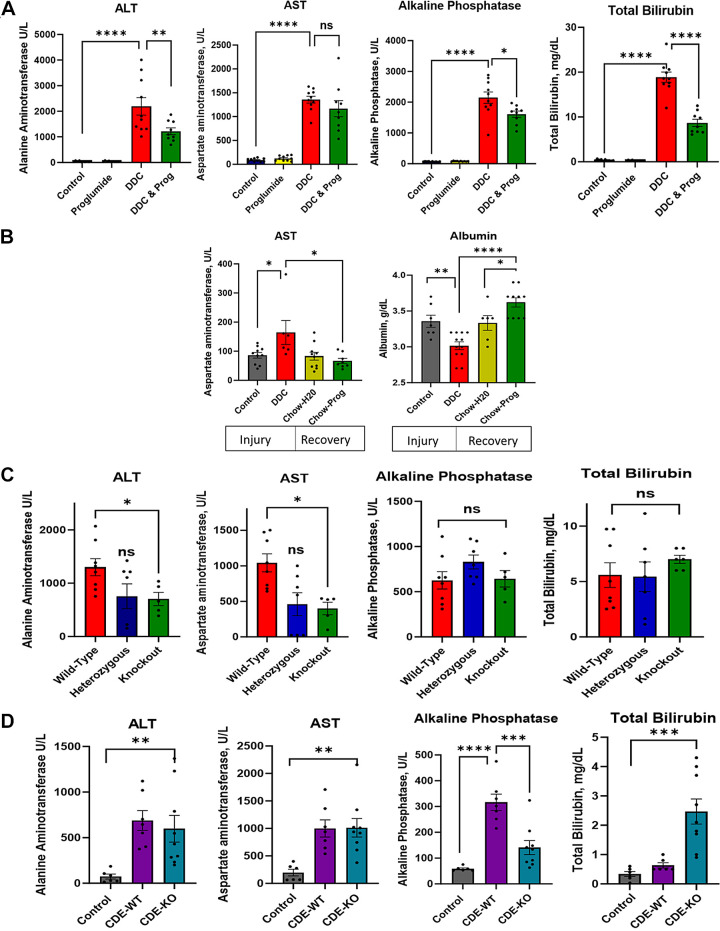
Evaluation of serum liver chemistries from mice on the DDC or CDE diets. *A*: pharmacological prevention study shows that the DDC diet increases serum hepatic transaminases, alkaline phosphatase, and total bilirubin in wild-type C57BL/6 mice compared with control or proglumide-treated mice on standard chow. These biochemical abnormalities are decreased in DDC-fed wild-type mice with the concomitant treatment with proglumide in the drinking water. *B*: proglumide-supplemented drinking water reverses liver injury and restores serum transaminase and albumin to normal levels in 4 wk in wild-type mice with established liver injury after being fed the DDC diet for 6 wk. *C*: CCK-BR-KO transgenic mice fed the DDC liver injury diet have significantly lower transaminases compared with wild-type mice on the same DDC diet. The cholestatic serum markers (alkaline phosphatase and total bilirubin) are similar in DDC-fed CCK-BR-KO mice and the wild-type DDC-fed mice. *D*: serum transaminases are increased in both wild-type and CCK-BR-KO mice fed the CDE-NASH-inducing diet compared with control mice on a standard chow diet. Serum alkaline phosphatase is significantly reduced in CCK-BR-KO mice fed the CDE diet compared with wild-type mice. Two-way ANOVA was performed. Columns on graphs with individual data points represent the means ± SE. ns, not significant; *P<0.05; **P <0.01; ***P <0.005; and ****P <0.001. ALT, alanine aminotransferase; AST, aspartate aminotransferase; CCK-BR, cholecystokinin-B receptor; CDE, choline-deficient ethionine; DDC, 3, 5-diethoxy-carbonyl 1,4-dihydrocollidine.

In this investigation, the liver DDC (0.1%) and the CDE (75%) injury diets were used, and the diets were studied in wild-type C57BL/6 mice as well as in a genetically engineered transgenic CCK-BR-KO mouse model. The four separate studies that were performed using these models are outlined in Supplemental Table S2, the study design for each of the four experiments is shown in Supplemental Fig. S1, and the measurement of animal weights and food consumption are shown in Supplemental Fig. S2. Biochemical evidence of liver injury was observed in the wild-type C57BL/6 mice fed the DDC liver injury diet with significant elevation in serum ALT and AST compared with mice on control diets (Fig. 1*A*). The DDC diet also induced cholestasis in the wild-type mice with elevation of serum alkaline phosphatase and total bilirubin. Mice on the DDC-fed diet that concomitantly received proglumide-treated drinking water showed a protective effect with the CCK-BR antagonist with lower serum transaminases, alkaline phosphatase, and total bilirubin despite the liver injury diet (Fig. 1*A*). Most human subjects, however, are diagnosed in a later stage with fibrosis and even cirrhosis after long term chronic hepatic inflammation from viral hepatitis or alcoholic liver disease. To determine if proglumide would still be beneficial in reducing liver inflammation and improving synthetic function after histologic damage had already occurred, we used another cohort of DDC-fed wild-type mice to induce liver fibrosis and inflammation. In this second study, elevation of transaminases and decrease in serum albumin were confirmed after 6 wk on the DDC diet (Fig. 1*B*) before the mice were placed on proglumide-supplemented drinking water or untreated drinking water for four subsequent weeks. Mice with established liver injury and fibrosis in this second study treated with proglumide showed decreased serum AST and improved serum albumin compared with those mice receiving untreated drinking water (Fig. 1*B*). These results suggest that proglumide has both a protective effect (preventive effect) for liver injury and can also reverse inflammation and improve liver synthetic function in the damaged liver. To elucidate whether the protective effects of proglumide on liver injury were a result of antagonism at the CCK-BR or due to a nonreceptor-mediated effect, we examined the serum biochemical changes in DDC-fed mice that were genetically engineered to lack the CCK-BR or CCK-BR-KO mice. In this third study, DDC-fed mice with knockout of the CCK-BR had significantly lower serum transaminases (ALT and AST) compared with wild-type mice on the same DDC injury diet (Fig. 1*C*). Mice that were heterozygous for the CCK-BR showed a partial effect with lower hepatic transaminases compared with control mice, but this difference was not statistically significant. In contrast to the proglumide-treated mice, the CCK-BR-KO or heterozygous mice did not have protection from the cholestatic changes, as exhibited by levels of alkaline phosphatase and total bilirubin comparable with that of DDC-fed wild-type mice (Fig. 1*C*). In the fourth study, wild-type mice and CCK-BR-KO mice were fed the NASH-inducing CDE diet, and biochemical injury was compared with control mice on standard chow. Liver transaminases were significantly increased in wild-type mice fed the CDE-NASH-inducing diet; however, the absence of the CCK-BR did not protect against this injury (Fig. 1*D*). In our prior work, proglumide significantly decreased liver transaminase elevation in CDE-fed mice (20); therefore, proglumide may have protective properties in the NASH injury model independent of the CCK-BR. Alkaline phosphate was lower in CDE-fed CCK-BR-KO mice. Total bilirubin was increased in CDE-fed CCK-BR-KO mice. However, this elevation was thought possibly to be due to hemolysis and difficulty obtaining the blood specimen in a few mice.

### Proglumide Therapy Decreases the Liver Mass Index of DDC-Fed Mice

The livers were dark due to cholestasis in the DDC-fed mice (Supplemental Fig. S3). The liver weight per gram of mouse weight (liver mass index) was calculated and showed that the liver mass index was significantly increased by 31% in DDC-fed mice (*P* < 0.0001) compared with control mice (Supplemental Fig. S4). Concomitant proglumide (combo) treatment significantly lowered the liver mass index (*P* = 0.02) in mice fed DDC that received proglumide-supplemented water (Supplemental Fig. S4).

### CCK-BR Blockade or Knockout Decreases Liver Fibrosis

**Figure 2.**
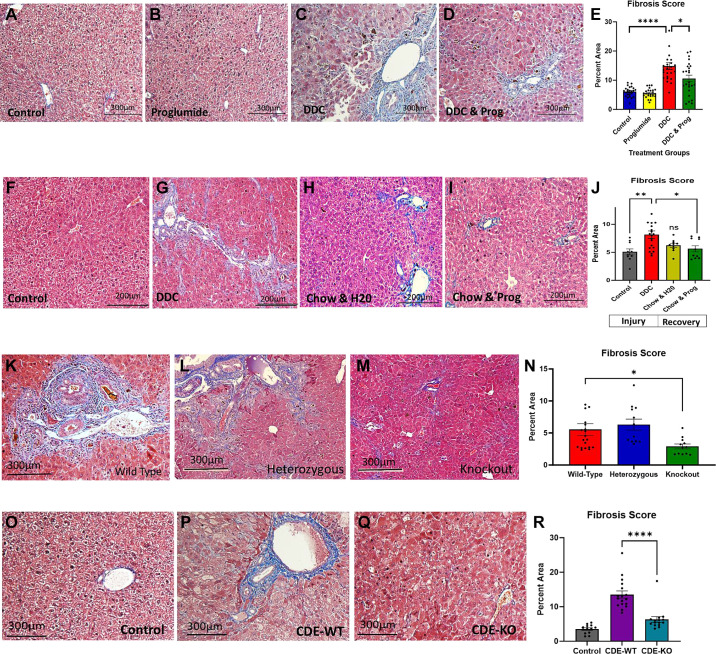
Evaluation of hepatic fibrosis by Masson’s trichrome stain in mouse livers. *A–E*: wild-type DDC-fed mice show increased fibrosis compared with controls. Proglumide significantly reduces fibrosis in DDC-fed mice. The graph shows means ± SE of fibrosis area from *n* = 25 images/group. *F–J*: from the reversal study, wild-type DDC-fed mice have increased fibrosis compared with controls. These mice were then treated with proglumide-supplemented water or untreated water and given standard chow. Proglumide-treated mice showed a significant reversal of fibrosis compared with mice on untreated water. Graph shows the fibrosis scores in the livers of mice after the 6-wk injury induction followed by the means ± SE scores during recovery when treated with water or proglumide. *K–N*: representative images of Masson’s trichrome stains from DDC-fed wild-type mice compared with DDC-fed heterozygous or CCK-BR-KO transgenic mice. Graph shows means ± SE of fibrosis area from *n* = 20 images/group. *O–R*: representative images of Masson’s trichrome stains from CDE-fed wild-type mice compared with CDE-fed heterozygous or CCK-BR-KO mice. Graph shows means ± SE of fibrosis area from *n* = 15 images/group. Significance was determined by two-way ANOVA was performed. Bar graphs with individual data points represent the means ± SE. ns, not significant; *P < 0.05; **P < 0.01; and ****P < 0.001. CCK-BR, cholecystokinin-B receptor; CDE, choline-deficient ethionine; DDC, 3, 5-diethoxy-carbonyl 1,4-dihydrocollidine.

Fibrosis is the result of years of chronic injury and inflammation. The etiology of fibrosis is now well recognized and results from the activation of tissue myofibroblasts or stellate cells that proliferate, migrate, and produce extracellular matrix (ECM) components, such as type I, III, and IV collagen, and express cytokines and chemokines (4, 29, 30). The hepatic stellate cell (HSC) undergoes morphological changes associated with the function and secretion of soluble factors that potentiate the inflammatory process (31). Since we previously found that proglumide therapy prevented the development of dysplastic nodules and HCC in CDE-fed mice (20) and because fibrosis is the greatest risk factor for the development of HCC (32), we examined the role of the CCK-BR on hepatic fibrosis using the DDC and CDE diets. When compared with livers of wild-type control mice on standard chow (Fig. 2*A*) or mice on standard chow with proglumide-treated water (Fig. 2*B*), livers of the wild-type mice fed the DDC diet exhibited marked hepatic fibrosis by Masson’s trichrome stain (Fig. 2*C*). Proglumide therapy significantly reduced hepatic fibrosis in wild-type mice on the DDC diet (Fig. 2*D*). Assessment of fibrosis in the liver histologic tissue sections was determined by quantitative analysis software that measured the percentage of the liver area with fibrosis from *n* = 25 images for each treatment group. Means ± SE fibrosis scores and individual data points are shown in Fig. 2*E.* In the reversal study after hepatic fibrosis with the DDC liver injury model was established and confirmed histologically in wild-type mice (Fig. 2*G*) compared with control standard chow-fed mice (Fig. 2*F*), a group of DDC-fed liver fibrosis mice received standard chow for four subsequent weeks and with proglumide-treated or untreated water. When compared with baseline fibrosis scores at *week 6* on the DDC diet, proglumide-treated mice had 72% less fibrosis during the 4-wk recovery period (Fig. 2*I*), and the mice receiving untreated water during the recovery period showed a 54% decline in fibrosis (Fig. 2*H*). The improvement in fibrosis area between the proglumide and water control mice was statistically significant (Fig. 2*J*) suggesting that proglumide expedited reversal of hepatic fibrosis after injury. Similarly, liver fibrosis was significantly less in DDC-fed CCK-BR-KO mice (Fig. 2*M*) compared with DDC-fed wild-type (Fig. 2*K*) or heterozygous mice (Fig. 2*L*) in the transgenic model *study C*. This difference was quantitatively significant in the histologic area analysis (Fig. 2*N*). The CDE-fed wild-type mice also exhibited increased fibrosis of the liver parenchyma (Fig. 2*P*) compared with standard chow-fed controls (Fig. 2*O*). In contrast, CDE-fed CCK-BR-KO mice had significantly less fibrosis than wild-type mice (Fig. 2, *Q* and *R*). Taken together these results imply that activation of the CCK-BR is involved in hepatic fibrosis in both the DDC diet and the CDE diet liver injury models. The importance of this finding is that not only did proglumide prevent fibrosis during liver injury, but it also reversed the fibrosis after it was established. This finding may be important to decrease the incidence of HCC by reducing the major risk factor, i.e., fibrosis.

### Hepatic Stem Cells Express CCK-BRs

**Figure 3.**
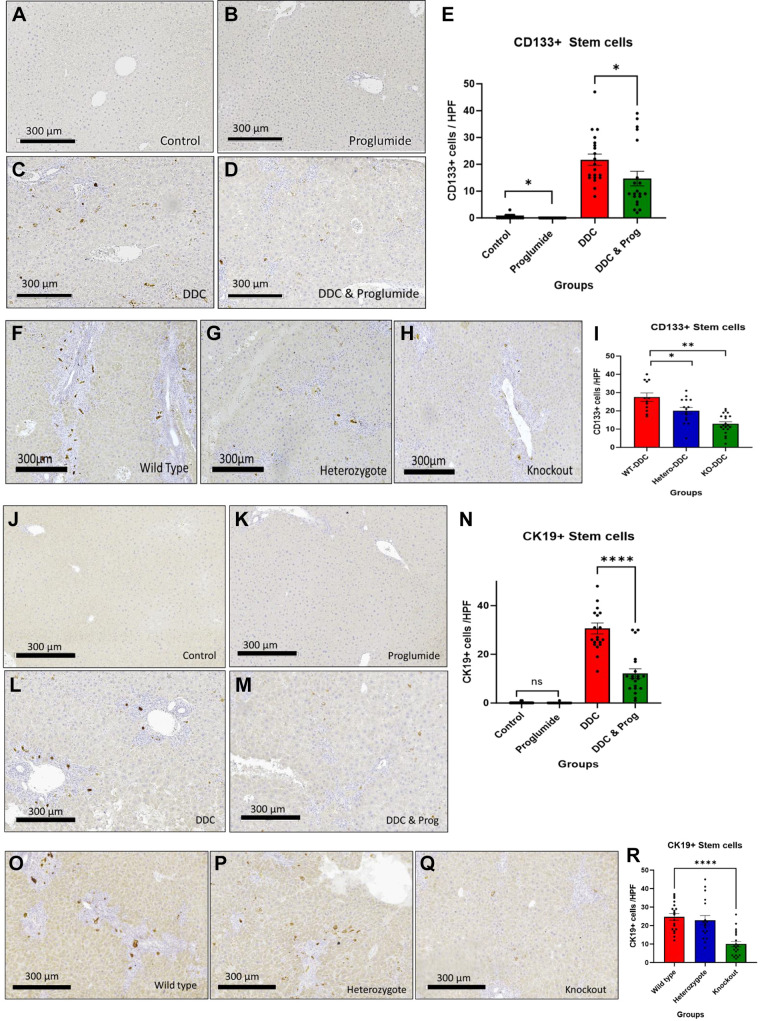
Activation of hepatic CD133+ and CK19+ stem cells requires the CCK-BR. *A*: representative images from sections of CD133+ stained liver sections from wild-type mice on standard chow show few, if any, CD133+ stem cells. *B*: image from control standard chow-fed mouse liver receiving proglumide-treated water shows few if any CD133+ stem cells. *C*: CD133 immunoreactivity is significantly increased in livers of DDC-fed wild-type mice per high-powered field. *D*: proglumide treatment decreases the number of CD133+ cells in the livers of DDC-fed wild-type mice. *E*: columns represent the means ± SE of CD133+ liver stem cells from (*n* = 20) liver section images of each treatment group in *study A*, a pharmacologic prevention study. *F*: representative image of CD133-stained liver sections from DDC-fed mice show increased CD133+ cells in DDC wild-type mice in *study C*, a genetically engineered model. *G*: image of a liver of a DDC-fed CCK-BR heterozygous mouse reacted with CD133 antibody. *H*: representative image from the liver of a DDC-fed CCK-BR-KO mouse shows a paucity of CD133+ immunoreactive cells. *I*: columns represent the means ± SE of CD133+ liver stem cells from (*n* = 20) liver section images of each treatment group in the genetically engineered mouse model *study C*. *J*: representative image from sections of CK19+ stained liver sections from wild-type mice on standard chow show few, in any CK19+ stem cells. *K*: image from control standard chow-fed mouse liver receiving proglumide-treated water shows few if any CK19+stem cells. *L*: CK19+ immunoreactivity is increased in the livers of DDC-fed wild-type mice per high-powered field. *M*: proglumide treatment decreases the number of CK19+ cells in the livers of DDC-fed wild-type mice. *N*: columns represent the means ± SE of CK19+ liver stem cells from (*n* = 20) liver section images of each treatment group in *study A*, a pharmacologic prevention study. *O*: representative image of CK19-stained liver sections from DDC-fed mice show increased CK19+ cells in DDC wild-type mice in *study C*, a genetically engineered model. *P*: image of a liver of a DDC-fed CCK-BR heterozygous mouse reacted with CK19 antibody. *Q*: representative image from the liver of a DDC-fed CCK-BR-KO mouse shows a paucity of CK19+ immunoreactive hepatic stem cells. *R*: columns represent the means ± SE of CD133+ liver stem cells from (*n* = 20) liver section images of each treatment group in the genetically engineered mouse model *study C*. Significance was determined by two-way ANOVA. Bar graphs with individual data points represent the means ± SE. ns, not significant; *P < 0.05; **P <0.01; and ****P <0.001, scale bar: 300 µm. CCK-BR, cholecystokinin-B receptor; DDC, 3, 5-diethoxy-carbonyl 1,4-dihydrocollidine.

Next, we investigated the expression of CCK-BRs in hepatic stem cells and the effects of proglumide therapy or CCK-BR-KO on stem cell activation and number in the liver injury mouse models. We analyzed stem cell numbers in liver histological sections using two separate immunohistochemical markers: CD133 and CK19. Both liver injury diets activated the expression of CD133+ stem cells, although the number was greater in the DDC-fed mice than the CDE-fed mice. In the wild-type mouse livers, there was minimal immunoreactivity for CD133+ cells in the liver sections of the control mice on standard chow (Fig. 3*A*) or standard chow with proglumide-treated water (Fig. 3*B*). CD133+ cells significantly increased in the livers of DDC-fed wild-type mice (Fig. 3*C*), and the mean number of CD133+ stem cells per high-powered field was significantly decreased in the livers of DDC-fed wild-type mice concomitantly treated with proglumide-supplemented water (Fig. 3*D*). The graph in Fig. 3*E* shows the means ± SE for each group with individual data points (*n* = 20/group). Representative images of CD133+ hepatic stem cells from the livers of DDC-fed mice in *study C* comparing wild-type DDC-fed mice to the DDC-fed transgenic model are shown in Figs. 3, *F–H*. The mean number of CD133+ cells in the liver sections per high-powered field (∼*n* = 25–27) in the DDC-fed wild-type mice (Fig. 3*F*) was comparable with the number of CD133+ cells in the DDC-fed wild-type mice from the prevention pharmacologic *study A* (Fig. 3*C*), confirming the reproducibility of our model. Livers of heterozygous DDC-fed mice exhibited fewer CD133+ stem cells (Fig. 3*G*) compared with wild-type DDC-fed mouse livers. Liver sections of the DDC-fed CCK-BR-KO mice had less than half of the CD133+ cells compared with wild-type mice (Fig. 3, *H* and *I*).

Representative images from the livers of CDE-fed mice that reacted with the CD133 antibody are shown in Supplemental Fig. S5. Compared with mice on the standard chow diet, the CDE diet increased the expression of CD133+ hepatic stem cells more than eightfold (Supplemental Fig. S5, *A* and *B*); however, the number of immunoreactive cells was about half that of the DDC-fed mice. The number of stem cells in the liver sections of CDE-fed mice was reduced by ∼50% in CCK-BR-KO mice (Supplemental Fig. S5, *C* and *D*) compared with CDE-fed mouse livers. These data suggest that the CCK-BR is in part responsible for the expression of CD133+ stem cells since both the pharmacologic receptor blockade and genetic downregulation of the CCK-BR decrease the number of hepatic stem cells.

Similar results were found in liver sections from the DDC-fed mice stained for another stem cell marker cytokeratin-19 (CK19). CK19+ cells were significantly increased in number in wild-type mice fed the DDC diet. Representative images from CK19-stained liver sections of control (Fig. 3, *J* and *K*) and DDC-fed mice are shown (Fig. 3*L*). Proglumide treatment reduced the number of CK19+ immunoreactive cells in the livers of the wild-type DDC-fed mice (Fig. 3, *M* and *N*). When compared with wild-type DDC-fed mice (Fig. 3*O*) CCK-BR-KO DDC-fed mice had significantly fewer immunoreactive CK19+ hepatic stem cells per high-powered field (Fig. 3, *Q* and *R*), and no difference was observed in the CK19 staining of the heterozygous mice (Fig. 3*P*) compared with the wild-type DDC-fed mouse livers. Taken together, the results from the CK19 immunohistochemistry support the CD133+ stem cell analysis showing that liver injury models induce the expression of hepatic stem cells. Both pharmacologic blockade of the CCK-BR with proglumide or genetic knockout of the CCK-BR decreases the number of immunoreactive hepatic stem cells in mouse liver injury models suggesting that the CCK-BR is involved in hepatic stem cell activation with liver injury.

### Hepatic Stem Cells Express CCK-BR

**Figure 4.**
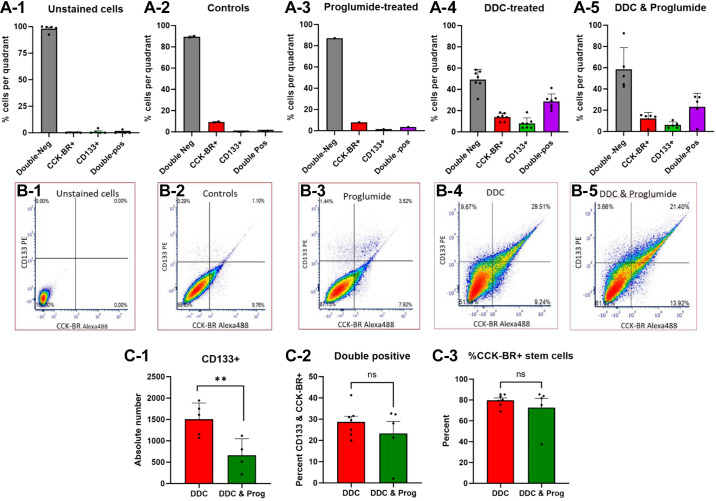
Hepatic stem cells express CCK-BRs by flow cytometry. *A-1* to *A-5*: columns represent means ± SE from each gated quadrant showing the percentage of cells from the dissociated livers that are double negative, CCK-BR+ cells, CD133+ cells, or double-positive cells (stain for both CCK-BR and CD133 surface receptors) in up to *n* = 7 experiments in each of the four cohorts of wild-type mice in the pharmacologic *study B*. Percentage of double-negative cells is around 90% in the cells from the standard chow-fed control mice. Percentage of CD133 immunoreactive cells significantly increases in dissociated cells from DDC-fed mice. *B-1* to *B-5*: representative flow cytometry figures of dissociated cells from livers of each group are shown. *B-1* shows unstained cells from DDC-fed mouse livers. *B-2* shows a flow image from control mouse livers fed standard chow. *B-3* shows a flow cytometry image of dissociated cells from the livers of standard chow-fed, proglumide-treated mice with the absence of stem cells or CCK-BRs. *B-4* is a flow cytometry image of liver cells from DDC-fed mice showing increased expression of CD133+ stem cells and over 78% of these express CCK-BRs. *B-5* shows an image of DDC-fed mice treated with proglumide with fewer CD133+ cells expressed but these cells also coexpress the CCK-BR. *C-1*: figure demonstrating the absolute number of CD133+ expressing cells by flow cytometry in DDC-fed mice is significantly decreased by cotreatment with proglumide. *C-2*: percentage of double-positive staining cells is the same in DDC-fed mice with and without proglumide-treated drinking water. *C-3*: percentage of CCK-BR-expressing cells among the steam cells is about 80% in DDC-fed mice and proglumide treatment does not alter the expression of the CCK-BR. Significance was determined by a two-way Student’s *t* test. Bar graphs with individual data points represent the means ± SE. ns, not significant; **P < 0.01. CCK-BR, cholecystokinin-B receptor; DDC, 3, 5-diethoxy-carbonyl 1,4-dihydrocollidine.

**Figure 5.**
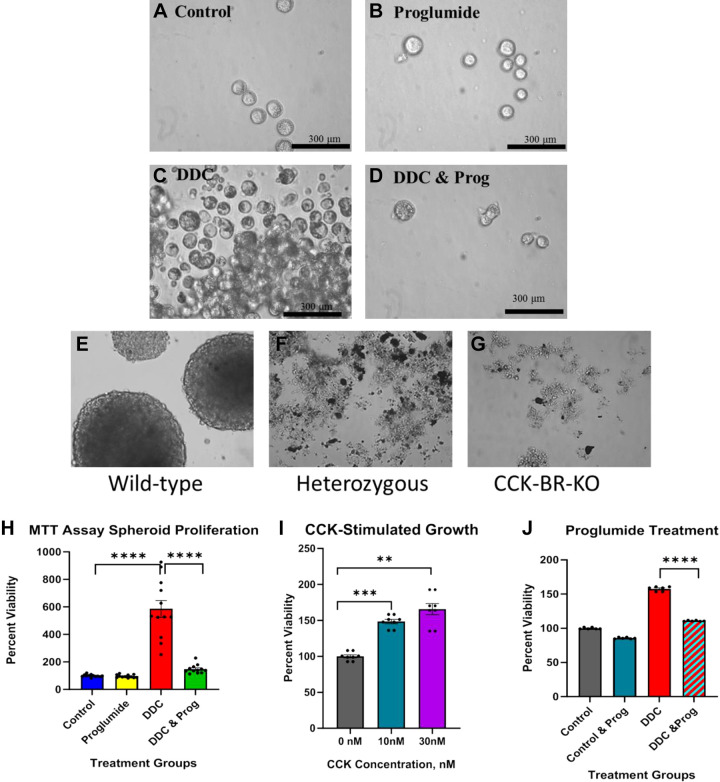
The formation of 3-D tumorspheres from livers is mediated by the CCK-BR. *A*: cells from livers of control mice on standard chow fail to form extensive tumorspheres. *B*: cells from dissociated livers of proglumide-alone-treated mice on a standard chow diet do not form many tumorspheres. *C*: cells from livers of DDC-fed wild-type mice form numerous tumorspheres that proliferate in cell culture (magnification ×4). *D*: liver cells isolated from wild-type mice on the DDC diet treated with proglumide water do not form many tumorspheres. *E*: higher magnification (magnification ×10) of tumorspheres formed from DDC-fed wild-type mice. *F*: dissociated cells from livers of DDC-fed heterozygous mice fail to form tumorspheres. *G*: cells from CCK-BR-KO mice livers fail to form tumorspheres. *H*: MTT proliferation of tumorspheres from each cohort of wild-type mice shows that only tumorspheres from the livers of DDC-fed mice proliferate and this effect is blocked with proglumide. *I*: treatment of tumorspheres from the liver cells of the wild-type DDC-fed mice increase in number with the addition of CCK (10 or 30 nM) to the growth media. *J*: treatment of tumorspheres from the liver cells of the wild-type DDC-fed mice with proglumide significantly decreases the proliferation. Significance is determined by two-way ANOVA. Bar graphs with individual data points represent the means ± SE. **P < 0.01; ***P <0.005; and ****P <0.001. CCK-BR, cholecystokinin-B receptor; DDC, 3, 5-diethoxy-carbonyl 1,4-dihydrocollidine; 3-D, three-dimensional.

Sections of livers from wild-type mice were dissociated with a Miltenyi apparatus with CD133 magnetic bead isolation to collect enriched samples of hepatic stem cells. These samples were then subjected to flow cytometry to analyze whether the CD133 cells were positive for the CCK-BR. Up to seven different mouse liver samples per group underwent liver dissociation and flow cytometry from DDC-fed mice with or without proglumide, or from standard chow-fed mice (controls) and with or without proglumide-treated water. Unstained cells used for gating are shown in Fig. 4*A-1*. Then, dissociated cells were incubated with a DyLight488-labeled CCK-BR antibody or a PE-labeled CD133 antibody to analyze cell surface receptors. The percentage of cells from each gated quadrant is shown for livers from control chow mice (Fig. 4*A-2*), control chow-fed mice with proglumide-treated water (Fig. 4*A-3*), DDC-fed mouse livers with untreated water (Fig. 4*A-4*), and DDC-fed mice with proglumide-treated water (Fig. 4*A-5*). About 90% of the cells from the livers of mice on standard chow in the control and proglumide-treated groups were double negative, implying very few if any CD133+ or CCK-BR+ immunoreactive cells. In mice on the DDC-fed diet, the percentage of CD133 and CCK-BR-positive cells increased. Corresponding representative flow images from each group are shown in Fig. 4, *B-1–B-5*.

The total number of CD133+ cells was reduced by 56% in the dissociated livers of DDC-fed mice treated with proglumide water (Fig. 4*C-1*) compared with DDC-fed mice on untreated water. No significant difference was observed between the percentage of double-positive stained cells in the DDC-fed mice whether on untreated water or with proglumide-treated water (Fig. 4*C-2*). About 35%–40% of the cells collected from dissociated livers of DDC-fed mice, expressed CD133 receptors, ∼78% of these costained for the CCK-BR (Fig. 4*C-3*). The flow data confirm that the DDC diet activates the expression of CD133+ stem cells in the mouse liver and the majority of these liver stem cells also express CCK-BRs. Proglumide does not change the CCK-BR expression on the stem cells but decreases the overall number of CD133+ stem cells with the liver injury diet.

Liver sections were also dissociated from wild-type CDE-fed mice and analyzed by flow cytometry for CCK-BR expression. A representative flow cytometry image from a wild-type CDE-fed mouse liver stained with CD133 and CCK-BR antibodies shows 23.6% of the cells are CD133+ (stem cells) and of these 74% express CCK-B receptors (Supplemental Fig. S6). These results are supportive of the immunohistochemical staining of the liver sections and demonstrate that both the DDC and CDE liver injury diets increase hepatic stem cells and that the majority of these stem cells express CCK-BRs. Proglumide treatment decreases the overall number of stem cells in the liver-dissociated samples, but it does not decrease the percentage of stem cells that express the CCK-BR. These results provide evidence that liver stem cells express the CCK-BR, and this expression is significantly upregulated with liver injury. However, proglumide treatment did not decrease CCK-BR expression in liver stem cells. Given this result, we then hypothesized that CCK-BR blockade does not prevent the development of CCK-BR-expressing stem cells with injury, but it may prevent the proliferative and oncogenic capacity of these stem cells. To test this hypothesis, the isolated liver cells were then cultured in vitro as 3-D spheroids.

### CCK-BR Expression Is Required for Hepatic 3-Dimensional Tumorsphere Formation

The ability of cells to form 3-D structures as tumorspheres (spheroids) or organoids in vitro depends on the stemness of the cells. Since stem cells have progenitor characteristics and have been implicated in hepatic carcinogenesis, we examined the role of the CCK-BR in CD133-enriched dissociated liver cells according to their tumorsphere-forming potential.

Dissociated cells from the livers of wild-type control or proglumide-treated mice on standard chow diet failed to form large numbers of tumorspheres (Fig. 5, *A* and *B*, respectively). In contrast, DDC-fed mice formed well-structured tumorspheres that proliferated in size and number over ∼10 days (Fig. 5*C*). Proglumide treatment in the water of wild-type DDC-fed mice prevented the liver cells from forming tumorsphere structures (Fig. 5*D*). Similarly, DDC-fed, wild-type mice in the transgenic study also formed tumorspheres that proliferated in culture (Fig. 5*E*, higher magnification). In contrast, isolated liver cells from DDC-fed heterozygous (Fig. 5*F*) and CCK-BR-KO mice (Fig. 5*G*) failed to form 3-D-tumorsphere structures in vitro.

The tumorsphere proliferation potential was evaluated using the MTT assay in vitro from isolated tumorspheres from each of the four cohorts of wild-type mice. Tumorspheres from the livers of standard chow-fed control or proglumide-treated mice failed to proliferate significantly over 4 days in culture (Fig. 5*H*). In contrast, tumorspheres from the livers of DDC-fed mice increased more than sixfold over controls, and the proliferation was blocked by proglumide-supplemented drinking water (Fig. 5*H*) indicating that the CCK-BR mediated growth of the tumorspheres. Three-dimensional tumorspheres from livers of wild-type DDC-fed mice that were treated in culture with CCK peptide exhibited increased proliferation (Fig. 5*I*) providing further evidence that the CCK-BR is involved in tumorsphere growth. Finally, tumorspheres derived from the livers of wild-type DDC-fed mice that were subsequently treated with exogenous proglumide in vitro show that proglumide blocks the proliferation (Fig. 5*J*).

### Proglumide Therapy Decreases Expression of Hepatic Inflammatory Cytokines

**Figure 6.**
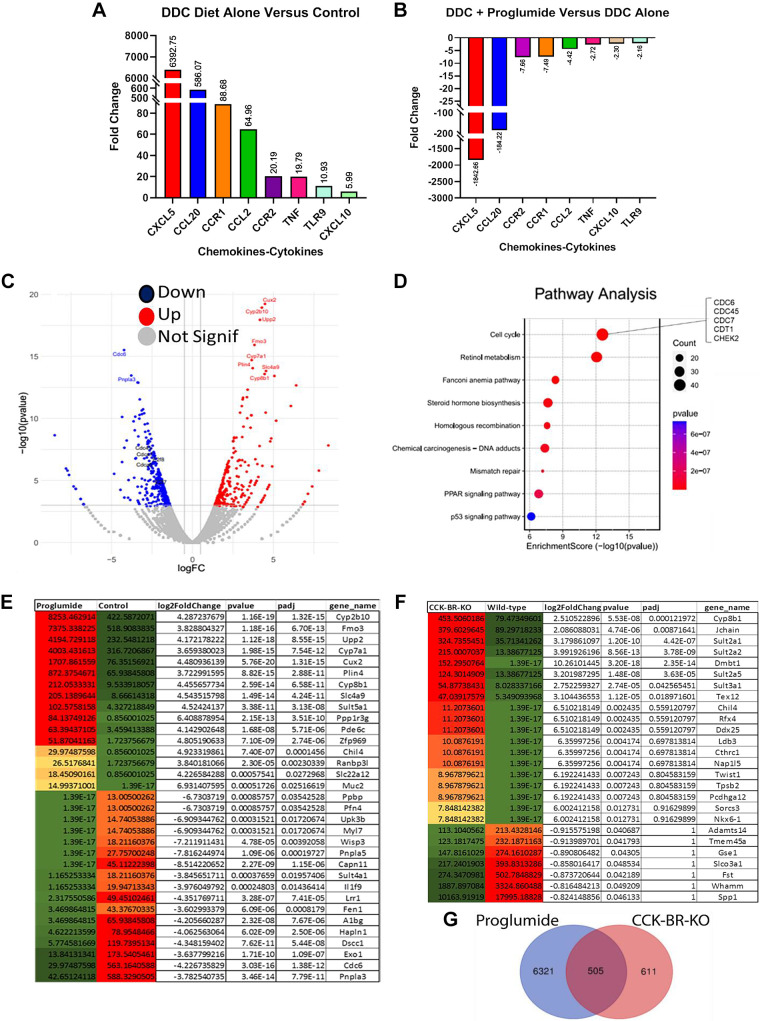
Differentially expressed genes by PCR and RNA sequencing. *A*: gene expression of cytokines and chemokines increased sixfold or more in livers of DDC-fed mice compared with controls. *B*: mRNA fold change in gene expression of chemokines and cytokines comparing DDC-fed mice on untreated H_2_O or proglumide-supplemented H_2_O is shown. *C*: volcano plot of RNAseq data from livers of mice treated with the 0.1% DDC diet and untreated H_2_O or proglumide-treated H_2_O. Downregulated genes with proglumide are in blue and upregulated genes with proglumide are in red. *D*: pathway enrichment analysis of genes analyzed with IPA software the Database for Annotation, Visualization, and Integrated Discovery (DAVID) shows specific pathways changed with proglumide therapy. *E*: heat map showing differentially expressed genes with fourfold change as determined by RNA sequencing of RNA from livers of DDC-fed mice compared with DDC-fed mice treated with proglumide. *F*: heat map showing differentially expressed genes as determined by RNA sequencing of RNA from livers of wild-type DDC-fed mice compared with RNA from livers of DDC-fed CCK-BR-KO mice. Upregulated genes are depicted in red and downregulated genes in green. *G*: Venn diagram of similar genes (*n* = 505) altered genes in livers of mice treated with proglumide and CCK-BR-KO mice. CCK-BR, cholecystokinin-B receptor; DDC, 3, 5-diethoxy-carbonyl 1,4-dihydrocollidine; IPA, ingenuity pathway analysis.

Liver inflammation is mediated by the activation of inflammatory cytokines and chemokines. Hepatic fibrosis results from the activation of tissue myofibroblasts or hepatic stellate cells that proliferate, migrate, express cytokines and chemokines, and produce extracellular matrix components, such as type I collagen (4). Using a liver RT^2^ Profiler PCR array, we examined the effects of the DDC injury diet and proglumide on the expression of mouse cancer inflammation and immunity cross talk genes. CxCL5 (33) and CCL20 (34) were highly over-expressed in the livers of the DDC-fed mice (Fig. 6*A*) followed by CCL1, CCL2, CCR2, TNF, TRL9, and CxCl10. In DDC-fed mice that were treated with proglumide, all these chemokines and cytokines decreased (Fig. 6*B*). CXCL5 was the most upregulated chemokine in the DDC injury model by PCR array, and proglumide decreased expression of CXCL5 more than 1,000-fold. CXCL5 has been identified as a crucial mediator downstream of the transcription protein sex-determining region Y-box 9 (Sox9) and since Sox9 is required for the self-renewal and tumor propagation of liver cancer stem cells (CSCs; 35); it has been characterized as a candidate CSC marker of HCC (36). Chemokine (C-C motif) ligand 20 (CCL20), also known as macrophage inflammatory protein (MIP)-3α and liver activation regulated chemokine (LARC), has been implicated in hepatic injury and development of HCC (37). CCL20 was another chemokine markedly upregulated in the DDC injury livers by PCR array and reversed with proglumide.

### Proglumide Alters Differentially Expressed Genes in Livers of DDC-Fed Mice

RNA sequencing of livers from DDC-fed mice and DDC-fed mice treated with proglumide revealed significant changes in differentially expressed genes. Figure 6*C* shows a volcano plot of significantly altered genes in the livers of DDC-fed mice that received proglumide-treated water compared with untreated water. Analysis of the differentially expressed genes (DEGs) from the RNA sequencing found that 296 genes were significantly upregulated (red dots) by proglumide therapy, and 332 genes were downregulated (blue dots) with proglumide. Figure 6*D* shows pathway enrichment analysis using the ingenuity pathway analysis (IPA) software and DAVID to analyze genes from RNAseq data in the DDC-fed mice with or without proglumide treatment. The cell cycle pathway was the most affected with significant alterations in genes involved in DNA replication (Fig. 6*D*).

A heat map of genes showing a fourfold or more change in RNA expression in the livers of mice on the DDC diet with proglumide therapy or on the DDC diet with untreated water is shown in Fig. 6*E.* Many of the genes that were upregulated with proglumide included those that coded for proteins or enzymes in the cytochrome P450 system, sulfotransferase, and ion transporters involved in hepatocyte and drug metabolism and detoxification. In particular, proglumide increased the expression of *CYP8B1*, a gene regulated by farnesyl-X receptor (FXR) and involved in bile acid synthesis (38). We previously showed that proglumide decreases NASH steatosis by interacting as a partial agonist at FXR (39). Many of the genes that were significantly downregulated in the livers of DDC-fed mice treated with proglumide were responsible for fibrosis (*Upk3b* and *HAPLN1*) (40, 41) or inflammation (*Il1f9*) (42). Other significantly downregulated genes by proglumide have been implicated as biomarkers in the progression to cirrhosis, such as *A1BG* (that codes for Alpha-1-B Glycoprotein; 43), *Ppbp* (that codes for proplatelet basic protein; 44), and *CDC6* (Cell Division Cycle 6; 45). Genes involved in hepatic stem cells and HCC development that were significantly downregulated in DDC-fed mice on proglumide included *Dscc1*, a putative HCC driver gene that promotes proliferation and is associated with poor prognosis in HCC (46), and *Capn11*, a member of the calpain family involved in HCC proliferation (47). Results from examination of the DEGs in the livers of DDC-fed mice treated with proglumide demonstrate that proglumide significantly downregulates the gene involved with hepatic fibrosis, inflammation, and oncogenesis.

### Effects of CCK-BR-KO on Gene Expression in Livers of DDC-Fed Mice

A heat map of genes identified by RNA sequencing from livers of DDC-fed wild-type or CCK-BR-KO mice that changed at least fourfold is shown in Fig. 6*F.* The most striking finding is that many genes that were significantly upregulated by knocking out the CCK-BR were tumor suppressor genes such as *Dmbt1* (48), *Pcdhga12* (protocadherin gamma; 49), *Sorcs3* (Sortilin related VPS10 domain-containing receptor 3; 50), and *Nkx6-1* (NK6 homeobox 1; 51). Most of the genes downregulated when the CCK-BR was knocked out promote HCC proliferation such as *Tmem45a* (transmembrane protein 45a; 52), *Spp1* (secreted phosphoprotein 1-Osteopontin; 53), *Slco3a1* (solute carrier organic anion transporter; 54), and *GSE1* (genetic suppressor element; 55). Knocking out the CCK-BR also decreased the expression of fibrosis-promoting genes including *Adamts14* (codes for a disintegrin-like and metallopeptidase; 56) and *Fst* (follistatin-like 1; 57). Five hundred and five genes were similarly altered in DDC-fed mice treated with proglumide or in transgenic CCK-BR-KO mice are shown in the Venn diagram (Fig. 6*G*). These results support that proglumide therapy mediates most of its mechanisms on inhibition of cell proliferation, fibrosis, and oncogenesis through the activation of the CCK-BR signaling pathway.

Since the RNA sequencing of DDC-fed mice with proglumide water compared with untreated water revealed changes in stem cells, we more thoroughly examined which genes involved in stem cell activation were affected by proglumide. Other differentially expressed genes implemented in hepatic stem cell activation and HCC oncogenesis affected by treatment with proglumide in DDC-fed mice are shown in Supplemental Fig. S7, *A*–*D*. In particular, genes downregulated with proglumide in DDC-fed mice that are involved with liver stem cell activation are shown in Supplemental Fig. S7*A* including several genes involved in the WNT/β-catenin pathway and genes implicated in the hedgehog pathway, such as *TPX2* and *FOXM1*. Several significant transcription factors (Supplemental Fig. S7*B*) were identified by RNA sequencing of DDC-fed mouse livers with or without proglumide. Differentially expressed genes implicated in cell cycle control (Supplemental Fig. S7*C*) that were downregulated in DDC-fed mice treated with proglumide support the antiproliferative effects of proglumide in the injured mouse liver. A heat map showing the upregulation and expression level of genes associated with HCC progression or HCC biomarkers in the livers of DDC-fed mice compared with DDC-fed mice with proglumide is shown in Supplemental Fig. S7*D*. Ingenuity pathway analysis (IPA) proposed regulatory network for DDC mice receiving proglumide treatment is shown in Supplemental Fig. S8. The IPA revealed predicted downregulation of cancer initiation, cell proliferation, and tumor formation in DDC-fed mice treated with proglumide.

### Proglumide Therapy Alters the Liver Microenvironment

**Figure 7.**
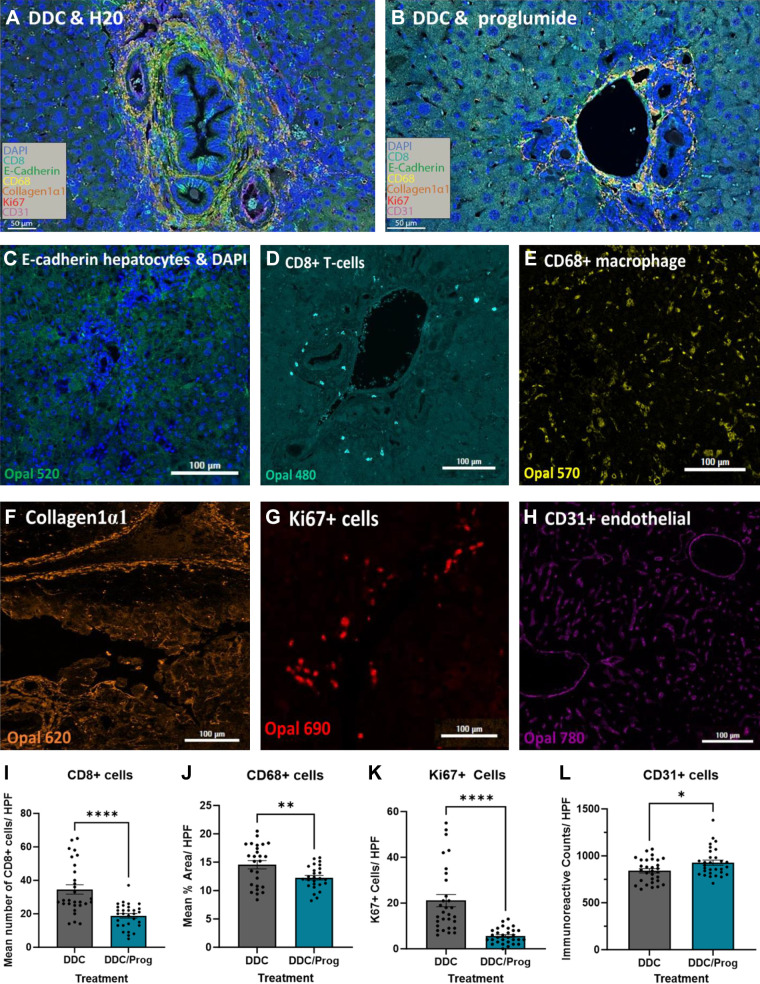
Immunohistochemistry of liver sections in DDC-fed and DDC-fed proglumide-treated mice as imaged by multiplex to study the liver microenvironment. *A*: representative image of a portal tract from a DDC-fed mouse shows extensive fibrosis and immune cell infiltrates. *B*: representative image of a liver portal tract from a DDC-fed mouse with proglumide treatment reveals less fibrosis and immune cell infiltrates. *C*: image from a liver showing immunoreactivity of hepatocytes reacted with E-cadherin (green) and the nuclei stained with DAPI (blue). *D*: immunohistochemistry of CD8+ T cells surrounding a portal tract (cyan). *E*: resident and infiltrating CD68+ macrophages in the liver sections of DDC-fed mice (yellow). *F*: staining of collagen1α1 in the liver periportal sites corresponds to fibrosis (orange). *G*: Ki67 proliferating cells are shown in the DDC liver with injury (red). *H*: vascular endothelium of the liver is identified with CD31 staining (purple). *I*: number of CD8+ cells per high-powered field (HPF) was decreased in DDC-fed mice livers treated with proglumide. *J*: density of CD68+ macrophages was also decreased in proglumide-treated mice. *K*: Ki67 immunoreactive proliferating cells were decreased in DDC-fed mouse livers when treated with proglumide. *L*: CD31+ vascular endothelium cells increased slightly in mice treated with proglumide. Significance was determined by analysis with Student’s *t* test: *P < 0.05; **P < 0.01; and ****P < 0.0001). DDC, 3, 5-diethoxy-carbonyl 1,4-dihydrocollidine.

Using multiplex immunohistochemistry, we compared liver sections from DDC-fed mice to liver sections of DDC-fed proglumide-treated mice to investigate changes and localization of immune cells, vasculature, collagen, and proliferating Ki67+ cells. A representative image from a hepatic portal tract in the DDC-fed mice with untreated water is shown in Fig. 7*A* compared with an image of a portal tract from a liver section of a DDC-fed proglumide-treated mouse (Fig. 7*B*). Representative images from individual channels are shown as captured on the PhenoChart imaging system. Figure 7*C* shows the blue-stained nuclei with DAPI and the hepatocytes in green reacted with E-cadherin for tissue localization. Although numerous cell types are involved in the liver microenvironment, we focused on two immune cell populations that are altered with liver injury. Figure 7*D* shows a representative image of CD8+ tissue infiltrating T lymphocytes localizing in the portal tracts. Immunoreactivity of resident Kupffer cells and activated macrophages are demonstrated with the CD68+ staining in Fig. 7*E* that was more diffuse throughout the liver parenchyma. Collagen1α1 (Fig. 7*F*) reacts with the fibrosis in the liver sections and strongly corroborates the fibrosis we observed with Masson’s trichrome stain. Ki67+ immunoreactive cells (Fig. 7*G*) are markers of proliferating liver cells. Our multiplex immunohistochemical analysis also focused on changes in the livers of DDC-fed mice treated with proglumide and association with the vasculature or liver sinusoidal endothelial cells (LSECs; Fig. 7*H*). Capillarization, characterized by defenestration, and the formation of a continuous basement membrane, is a common phenomenon in chronic liver diseases that occur in the LSECs and is often related to increasing hepatic fibrosis (58). Computer software analysis of the immunoreactivity comparing multiple sections and images of livers from DDC-treated mice to livers of DDC-fed proglumide-treated mice showed significant changes. In particular, CD8+ T lymphocytes that are associated with chronic hepatitis and inflammation were significantly decreased by 45% in the livers of proglumide-treated mice (Fig. 7*I*). Similarly, CD68+ macrophage density was also significantly decreased in the livers of DDC-fed mice treated with proglumide-supplemented water (Fig. 7*J*). Ki67+ proliferative index decreased by 74% in liver sections of DDC-fed mice treated with the CCK-BR antagonist proglumide (Fig. 7*K*). The number of CD31+ endothelial cells was increased with proglumide (Fig. 7*L*), suggesting preservation or improved sinusoidal blood flow. These data show that treatment of the mice with proglumide altered the liver microenvironment rendering it less oncogenic.

## DISCUSSION

Here, we identified a novel G protein-coupled receptor signaling pathway involved in hepatic stem cell activation and oncogenesis. Cholecystokinin-B receptors are not found in the normal liver but become expressed in liver injury and are overexpressed in HCC (13). Using flow cytometry, we confirmed that isolated CD133+ expressing stem cells also coexpress the CCK-BRs and that these cells increase in number with liver injury induced by a chemical or a NASH-inducing diet. The “stemness” of the cells isolated from the injured livers was confirmed by their ability to form 3-D-tumorspheres that proliferated in cell culture. Since the ability to form tumorspheres from dissociated and stem cell enriched liver cells was prevented when the mice were concomitantly treated with the CCK-BR antagonist proglumide or when mice were genetically engineered to be CCK-BR-null, this implies that the CCK-BR on the activated stem cells is involved with 3-D tumorsphere formation and liver oncogenesis. We previously showed that proglumide therapy could prevent the development of hepatic dysplastic nodules and HCC in mice fed a saturated fat diet (20). Although stem cells are implicated in the development of HCC, they are also important for hepatic regeneration after injury (6). Therefore, selective inhibition of the stem cell receptor involved in oncogenesis (e.g., the CCK-BR) rather than elimination of hepatic stem cells is important for liver recovery after an insult. Indeed, our work demonstrated when proglumide therapy was introduced to the mice after the injury with the DDC diet, recovery was hastened with improvement in hepatic transaminases, albumin, and fibrosis. The proliferative potential of the tumorspheres in culture through the CCK-BR was demonstrated by showing increased cell viability in the MTT assay after stimulation with a CCK-BR agonist cholecystokinin. Likewise, exogenous application of proglumide decreased the proliferation of tumorspheres in culture showing that blockade of the CCK-BR signaling pathway slows growth. Immunohistochemical staining for Ki67+ proliferative cells in sections of DDC-fed mouse livers confirmed that blockade of the CCK-BR pathway with proglumide prevented proliferation and oncogenic potential. These cumulative results support that the CCK-BR signaling pathway is involved in hepatic stem cell activation and hepatic oncogenesis after injury.

Another important finding in this work is that we demonstrated hepatic fibrosis is mediated in part by the CCK-BR. In both the proglumide-treated mice and the mice with genetic knockout of the CCK-BR, we demonstrated a decrease in hepatic fibrosis after two different liver injury diets. In the studies where proglumide was administered concomitantly with the injury diet (chemical injury or saturated fat), we showed that this therapy may help prevent fibrosis. Our finding that proglumide also reversed fibrosis after it is established histologically with the DDC injury diet is of even greater importance. For years it was thought that cirrhosis and fibrosis were not reversible; however, clinical studies have now shown that successful treatment of hepatitis C (59) and hepatitis B (60) can indeed reverse cirrhosis. Fibrosis is now well recognized to result from the activation of tissue myofibroblasts or hepatic stellate cells that proliferate, migrate, and produce extracellular matrix components, such as type I and type IV collagen, and express cytokines and chemokines (4, 29). The liver hepatic stellate cell (2) and the pancreatic stellate cells (61) are very similar and the normal quiescent stellate cells lose their lipid droplets upon activation and undergo morphological changes associated with function and secretion of soluble factors that potentiate the inflammatory process. Some of these functions and secretions include α-smooth muscle actin (α-SMA) expression, proliferation, extracellular matrix protein (ECM) production, cytokine and chemokine production, adhesion molecule (ICSM-1) expression, migration, contractility, matrix metalloproteinase (MMP) expression, toll-like receptor expression, and angiogenesis (4, 29). CCK-BRs have been reported on isolated rat and human pancreatic stellate cells and repeated CCK administration has been shown to activate the pancreatic stellate cells (62, 63). We recently showed that proglumide treatment decreased migration, proliferation, and collagen production in vitro in both mouse and human pancreatic stellate cells (64), and therefore, one mechanism that may be involved in the decreased hepatic fibrosis observed in this investigation includes receptor blockade or inactivation of the CCK receptor on hepatic stellate cells. In a recent Phase 1 safety and dose-finding clinical trial with proglumide in human subjects with nonalcoholic steatohepatitis, proglumide therapy over 12 wk decreased hepatic fibrosis scores by FibroScan, lowered serum hydroxyproline levels, and altered serum microRNAs associated with fibrosis (65). In support of our histologic findings of decreased hepatic fibrosis when blocking or genetically altering the CCK-BR, includes the findings of altered fibrosis-associated genes identified with the RNA sequencing analysis. In particular, *HAPLN1*, the gene that codes for hyaluronan and proteoglycan link protein 1, was downregulated more than fourfold by proglumide. Accumulation of hyaluronan in the liver and as a biomarker in the blood is associated with fibrosis and cirrhosis (41), and it may also serve as a signaling molecule to facilitate liver disease progression. In addition to hyaluronan being downregulated by proglumide, *HAS1* the gene that codes for hyaluronan synthase 1 is downregulated more than fivefold with proglumide in DDC-fed mouse livers. The expression of several collagen genes was also decreased with proglumide including collagen IV (66), collagen1α1 (67), and pro-collagen1 all of which are associated with hepatic fibrosis. Our investigation found that some of the changes in differentially expressed genes involved with hepatic steatosis, such as the farnesyl X receptor (FXR) and *PNPLA3*, were only altered by proglumide and not by genetic knockout of the CCK-BR. These results suggest that proglumide mediates its effects on hepatic steatosis by a non-CCK-BR mechanism. Supportive of the non-CCK-BR mechanism in hepatic steatosis includes our prior research showing that proglumide decreases hepatic steatosis by serving as a partial FXR agonist (39).

Interference or decreased signaling at the CCK-BR in both the pharmacologic and genetic models displayed decreased hepatic inflammation. The first evidence supporting a diminished inflammatory response includes decreased serum transaminases. The PCR array analysis from DDC-fed or DDC-fed proglumide-treated livers revealed significantly decreased expression in inflammatory cytokines and chemokines. Many of these inflammatory peptides are released from myofibroblasts and activated tissue macrophages to induce hepatocyte injury and recruit immune cells such as CD8+ T lymphocytes. CxCL5 and CCL20 were significantly downregulated with proglumide. *Il1f9* mRNA expression was downregulated fourfold by RNAseq in DDC-fed mouse livers treated with proglumide water; this gene codes for IL-36R, the fifth newly discovered cytokine of the interleukin (IL)-36 signaling family that induces inflammatory responses (42). The mechanism involved in decreased inflammation in the livers of mice treated with proglumide may represent cross talk between G protein-coupled receptors (GPCRs; 68). The CCK-BR and chemokine receptors both influenced the action of the other GPCRs, either by sensitizing or desensitizing the intracellular signaling or downstream pathways of each other or by forming heterodimers (69) to mediate the inflammatory effects.

One of the limitations of this investigation was that organoids and organoid-based xenograft models were not performed. Tumorspheres were cultured due to the backorder of Matrigel during the COVID-19 pandemic. For future experiments, hepatic stem cells isolated from injured livers and control mice could be cultured for organoids and then injected into mice for xenograft models. Tumor formation could be monitored; treatment with proglumide could be performed to assess CCK-BR blockade’s effect on tumor growth. Such experiments would provide further evidence of the CCK-BR’s role in liver stem cell oncogenesis and HCC formation following liver injury.

Another limitation was that the CDE-fed mice were on the NASH-inducing diet for only 8 wk rather than a longer period. Indeed, we previously showed that proglumide treatment ameliorated both liver injury markers and fibrosis in this 12-wk CDE wild-type mouse model (20). Future experiments with the CCK-BR-KO mice receiving the 12-wk CDE diet could be performed to determine whether the CCK-BR’s absence is protective in more advanced steatosis and HCC formation.

Changes in the liver microenvironment with liver injury were confirmed with RNAseq, PCR cytokine array, and immunoreactivity using Multiplex imaging. With all these modalities, interruption of the CCK-BR signaling pathway improved hepatic function while decreasing inflammatory response, fibrosis, and oncogenic potential. The CCK-BR is an important signaling pathway involved in hepatic injury and oncogenesis. Interruption of CCK-BR signaling either genetically or pharmacologically decreases and does not impede the liver’s physiologic ability to regenerate after injury but decreases the risk for oncogenesis. With the rising incidence of HCC worldwide, novel strategies to hasten liver recovery after an insult, reverse the preexisting damage, and prevent the risk for HCC development in those with hepatic fibrosis are of utmost importance. Proglumide therapy has the potential to reverse hepatic fibrosis and alter the liver microenvironment. Furthermore, this compound is clinically safe in human subjects with chronic liver injury (65).

## DATA AVAILABILITY

Data availability and requests for resources and reagents should be directed to and will be fulfilled by the lead contact, Dr. Martha D. Gay: mdg111@georgetown.edu.

## SUPPLEMENTAL DATA

10.5281/zenodo.10472291 Supplemental Figs. S1–S8 and Tables S1 and S2: https://doi.org/10.5281/zenodo.10472291.

## GRANTS

This work was supported by National Institutes of Health (NIH) Grant K01 Award CA255572 (to M.D.G.), Department of Defense Grant CA200380 (to J.P.S.), Special Research restart discretionary funding from Georgetown University MC Dean for Research (to N.S.), Funding from the graduate school program in Tumor Biology at Georgetown Lombardi Cancer Center (to A.Y. and Y.H.), and a Summer Undergraduate Research Fellowships from the American Physiological Society (to J.C.D.). These studies were conducted in part at the Georgetown Lombardi Comprehensive Cancer Center Histopathology & Tissue Culture, and Flow cytometry Shared resource, which is supported by NIH/NCI Grant P30-CA051008.

## DISCLOSURES

Georgetown University has an issued patent for proglumide in NASH and cancer and Jill P. Smith is an inventor of the intellectual property. None of the other authors has any conflicts of interest, financial or otherwise, to disclose.

## AUTHOR CONTRIBUTIONS

M.D.G., J.C.D., N.S., and J.P.S. conceived and designed research; M.D.G., J.C.D., W.C., Y.H., A.A.Y., T.D., N.S., and J.P.S. performed experiments; W.C., H.F., and J.P.S. analyzed data; M.D.G., J.C.D., W.C., Y.H., A.A.Y., T.D., H.F., N.S., and J.P.S. interpreted results of experiments; M.D.G., J.C.D., W.C., Y.H., A.A.Y., and J.P.S. prepared figures; M.D.G., J.C.D., and J.P.S. drafted manuscript; M.D.G., J.C.D., and J.P.S. edited and revised manuscript; M.D.G., J.C.D., W.C., Y.H., A.A.Y., T.D., H.F., N.S., and J.P.S. approved final version of manuscript.
